# Novel opto-fluidic drug delivery system for efficient cellular transfection

**DOI:** 10.1186/s12951-023-01797-3

**Published:** 2023-02-06

**Authors:** Majid Layachi, Anthony Treizebré, Laurent Hay, David Gilbert, Jean Pesez, Quentin D’Acremont, Kevin Braeckmans, Quentin Thommen, Emmanuel Courtade

**Affiliations:** 1 Laboratoire Physique des Lasers, Atomes et Molécules - UMR 8523, Université de Lille, 59655 Villeneuve d’Ascq, France; 2 Institut d’Électronique, de Microélectronique et de Nanotechnologie - UMR CNRS 8520, Université de Lille, 59655 Villeneuve d’Ascq, France; 3 Laboratory for General Biochemistry and Physical Pharmacy, Ghent University, 9000 Ghent, Belgium; 4 CANTHER - Cancer Heterogeneity Plasticity and Resistance to Therapies - UMR9020-UMR1277, Université de Lille, CNRS, Inserm, CHU Lille, Institut Pasteur de Lille, 59000 Lille, France; 5 Present Address: Laboratoire Charles Coulomb - UMR 5221, Université de Montpellier, Montpellier, France

**Keywords:** Photoporation, Vapour nanobubbles, Microfluidics, High-throughput intracellular delivery, Nanoparticle micro-positioning

## Abstract

Intracellular drug delivery is at the heart of many diagnosis procedures and a key step in gene therapy. Research has been conducted to bypass cell barriers for controlled intracellular drug release and made consistent progress. However, state-of-the-art techniques based on non-viral carriers or physical methods suffer several drawbacks, including limited delivery yield, low throughput or low viability, which are key parameters in therapeutics, diagnostics and drug delivery. Nevertheless, gold nanoparticle (AuNP) mediated photoporation has stood out as a promising approach to permeabilize cell membranes through laser induced Vapour NanoBubble (VNB) generation, allowing the influx of external cargo molecules into cells. However, its use as a transfection technology for the genetic manipulation of therapeutic cells is hindered by the presence of non-degradable gold nanoparticles. Here, we report a new optofluidic method bringing gold nanoparticles in close proximity to cells for photoporation, while avoiding direct contact with cells by taking advantage of hydrodynamic focusing in a multi-flow device. Cells were successfully photoporated with $\sim {70}{\%}$ efficiency with no significant reduction in cell viability at a throughput ranging from $10^3$ to $10^4~\text {cells}~{\hbox {min}^{-1}}$. This optofluidic approach provides prospects of translating photoporation from an R &D setting to clinical use for producing genetically engineered therapeutic cells.

## Background

Targeted and controlled delivery of molecular agents is firmly associated with diagnosis and therapy. Techniques were developed to achieve intracellular delivery of exogenous effector molecules with high transfection efficiency and cell viability. The most common strategies can be classified into vector based techniques and physical permeabilization techniques. Viral vectors have demonstrated high efficiency but are associated with significant risks such as immunogenicity and oncogenesis. Moreover, they have limited packaging capabilities of effector molecules [1]. Chemical vectors can be used instead but often perform poorly on primary and “hard-to-transfect” cell types [2, 3]. On the other hand, physical strategies were developed to permeate the cell membrane, allowing direct access to the cytosol [4]. Microinjection and electroporation, for example, achieve this goal by creating transient pores in the cell membrane. The high efficiency of the former and the high throughput of the latter are respectively balanced by low throughput, yield variability as well as high toxicity [5–7].

Alternatively, permeabilization of the cell membrane can be achieved by using light-matter interaction. For instance, when a gold nanoparticle (AuNP) is irradiated with a short laser pulse carrying adequate energy, Surface Plasmon Resonance (SPR) occurs resulting in an energy transfer to the surrounding medium in the form of heat or pressure waves [8, 9]. Heat dissipation is the dominant phenomenon at low laser intensities. When AuNPs are in contact with the cell membrane, it leads to permeabilization by phase transition or lipid bi-layer denaturation [10]. At higher intensities, the irradiation of AuNPs in an aqueous medium creates cavitating Vapour NanoBubbles (VNB) around the plasmonic nanostructures.

**Fig. 1:**
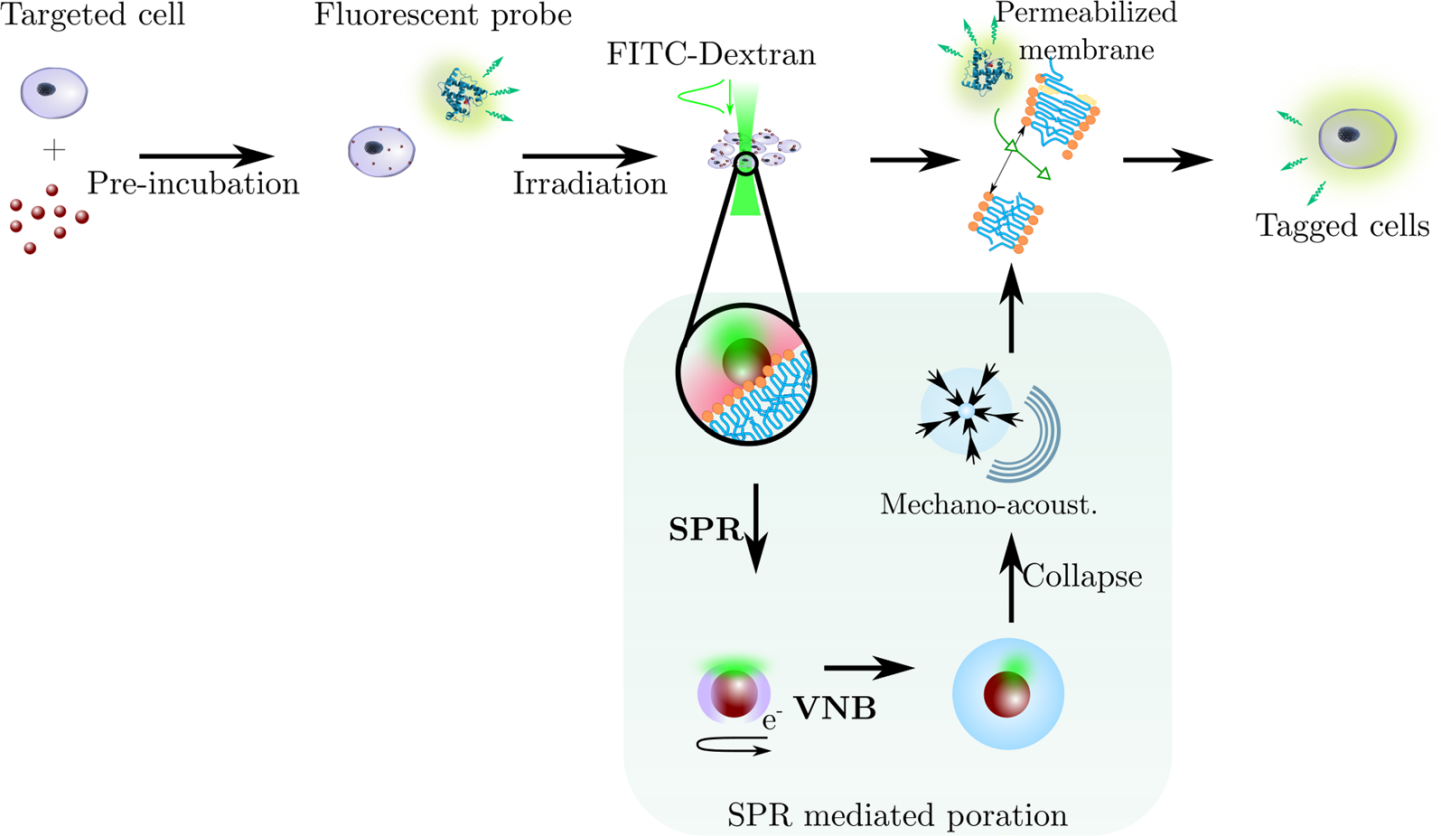
AuNP mediated photoporation: principle and biological application. AuNP-mediated adherent photoporation. ${70}~{\hbox {nm}}$ gold nanoparticles (AuNP) are attached to the cell membrane during a pre-incubation step of adherent HeLa cells and irradiated with a nanosecond laser pulse. The interaction between the metal electrons and the laser pulse results in Surface Plasmon Resonance (SPR). Depending on the fluence, the oscillatory movement of the excited electrons causes a rise of temperature or the creation of a Vapour NanoBubble (VNB). In the latter case and as a result to the bubble dynamic and its collapse, transient nanopores are thus generated in the cell membrane through which the exogenous fluorescent probe diffuses inside the cytoplasm. We obtain then FITC-labeled cells with the creation of AuNP debris.

The dynamics of these bubbles generate mechano-acoustic stress. Transient membrane nanopores can then be induced at the location where AuNPs are attached (Fig. 1). Because the pores are transient, efficient intracellular delivery of molecules present in the surrounding medium can be achieved while largely preserving cell viability [11, 12]. This method offers many advantages, namely controlling the number of pores by tuning the AuNP concentration, controlling the size of the created pores by tuning the laser intensity and controlling which cells are permeabilized by tuning the position of the laser beam [13, 14]. However, pre-incubation of cells with AuNPs is necessary to allow the particles to associate with the cells (Fig. 1). This step implies that cells are in contact with gold nanoparticles for a significant period of time. At the nanoscale, studies show variability in the toxicity response of cells exposed to gold nanostructures. This variability can be dependent not only on AuNP size, charge or concentration, but also on cell type and culture method [15–20]. Moreover, this direct contact between cells and AuNPs is a serious setback if treated cells are to be used *in-vivo*, such as for cell-based therapy. A work-around has been recently tested by seeding gold plasmonic patches or matrices in culture wells onto which cells can be cultured and irradiated [21–23]. Although good transfection rates were obtained, the preparation steps remain complex with no guarantee of uniformly distributed plasmonic nanostructures. Another solution to the potential toxicity due to gold nanoparticles is to replace them with biodegradable components. Using polydopamine nanoparticles as alternative sensitizers, good results were achieved on adherent and suspension cells - including hard to transfect human T-cells. Nevertheless, the nanostructures require to be attached to the cell membranes and therefore might be incorporated by the cells [24]. The mechano-acoustic effects induced by the laser generated VNBs have a range of action. Consequently, appropriately excited AuNPs in the VNB regime do not require a strong contact with the cell membrane to induce permeabilization [25, 26]. Hence, AuNPs suspended in the vicinity of cells are likely to produce membrane permeabilization without pre-incubation (Fig. 1). The use of microfluidics for cell handling presents many advantages especially in the field of cellular photoporation. It can provide non-specific and high throughput production of highly viable, healthy and potent transfected cell samples in a sterile manner with minimal human handling [27]. The use of microfluidics also allows a substantial reduction in sample and reagent volume due to a reduction in scale. Additionally, a laminar flow inherently allows high spatiotemporal flow control to fulfill the conditions for VNBs generation even at high flow rates [28]. Finally, the miniaturization inherently permits the integration of other modalities, for instance combining an integrated optofluidic cell isolation with a cargo delivery follow-up, opening new possibilities for fully automated systems [29–31]. The coupling of photoporation and microfluidics has already been tried thanks to the engineering of new plasmonic mediators such as fibers or clusters. The plasmonic components are grafted to the cells and remain in the vicinity of the cell membrane. Although $\le {90}{\%}$ transfection efficacy is obtained with ${2}~{\hbox {mg mL}^{-1}}$ solution of FITC-Dextran ${10}~{\hbox {kDa}}$, cytotoxicity remains an issue due to plasmonic debris inside the cytoplasm [32, 33].

In this paper, we report a new optofluidic method bringing gold nanoparticles in close proximity to cells for photoporation, while avoiding direct contact with cells through a flow-focusing configuration for spatio-temporal flow control. A mixture of HeLa WT cancer cells and Gold nanoparticles were injected in a multi-flow microfluidic chip at fixed flow rate and irradiated with a Q-switch laser. Cells were successfully photoporated with $\sim {70\pm 5}{\%}$ efficiency (assessed through the uptake FITC-Dextran ${10}~{\hbox {kDa}}$ using fluorescence microscopy) with no significant reduction in cell viability at a throughput ranging from $10^3$ to $10^4~\text {cells}~{\hbox {min}^{-1}}$. This optofluidic approach provides prospects of translating photoporation from an R&D setting to clinical use for producing genetically engineered therapeutic cells.

## Results and Discussions

### Optofluidic design for photoporation by VNB generation

**Fig. 2:**
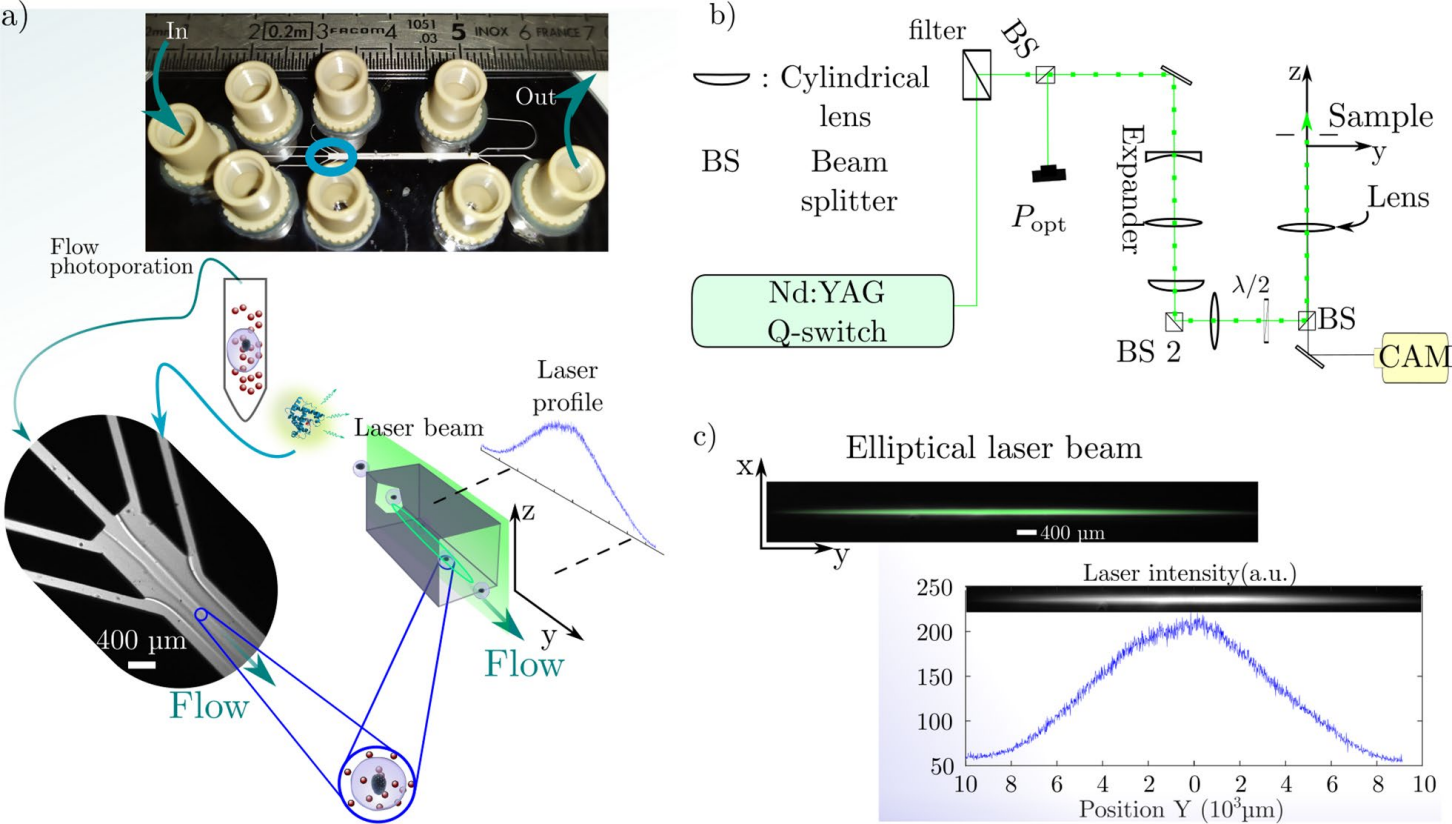
Adherent and Flow photoporation: experimental set-up and protocol. **a** Scheme for in-chip flow photoporation. Irradiation of a mix of suspended HeLa cells and AuNPs by the elliptical nanosecond laser pulse within a microfluidic chip with ${100}~{\upmu \hbox {m}}$ deep channels. The chip is an assembly of glass-silicon-glass obtained by anodic-bonding and dry-etching of the Si wafer. The two-stage flow focusing arranges the flow with respect to the elliptical laser beam. The suspension is injected at fixed flowrates with respect to the laser repetition rate along with FITC-Dextran solution at fixed concentration. Irradiated samples are retrieved at the outlet before washing and fluorescence microscopy. **b** Optical set-up to shape the nanosecond laser beam with respect to the microfluidic geometry. The same apparatus is used for both adherent and flow photoporation and produces a laser beam $\simeq {120}\upmu {\hbox {m}}$ wide in the *x* direction. **c** Elliptical laser beam at the sample. The fluence profile of the beam is measured by Rhodamine B fluorescence on the camera to obtain the local laser profile intensity value with respect to the position during the photoporation

The fluidic part of the device is a custom-made microfluidic chip to guarantee a laminar flow at high flow rates $\simeq {0.1}~{\hbox {mL min}^{-1}}$ to ${1}~{\hbox {mL min}^{-1}}$ (Fig. 2a). The chip consists of a Glass-Silicon-Glass assembly to sustain high pressure and insures good transparency for visible light. The flow is generated with a flow-rate regulated pressure unit. The geometry is a flow focusing design with a two stage sheath flow injection to organize the flow with respect to the velocity and shear stress profile within the main channel. The second part of the device is the optical apparatus (Fig. 2b). It consists of a Q-switch laser emitting nanosecond pulses at ${532}~{\hbox {nm}}$ with a ${10}~{\hbox {Hz}}$ repetition rate. To optimize the throughput, the circular beam is expanded in the flow direction to form and elliptical beam irradiating most of the channel with one pulse (Fig. 2c).

### VNB induced membrane nanopores in adherent cells

**Fig. 3:**
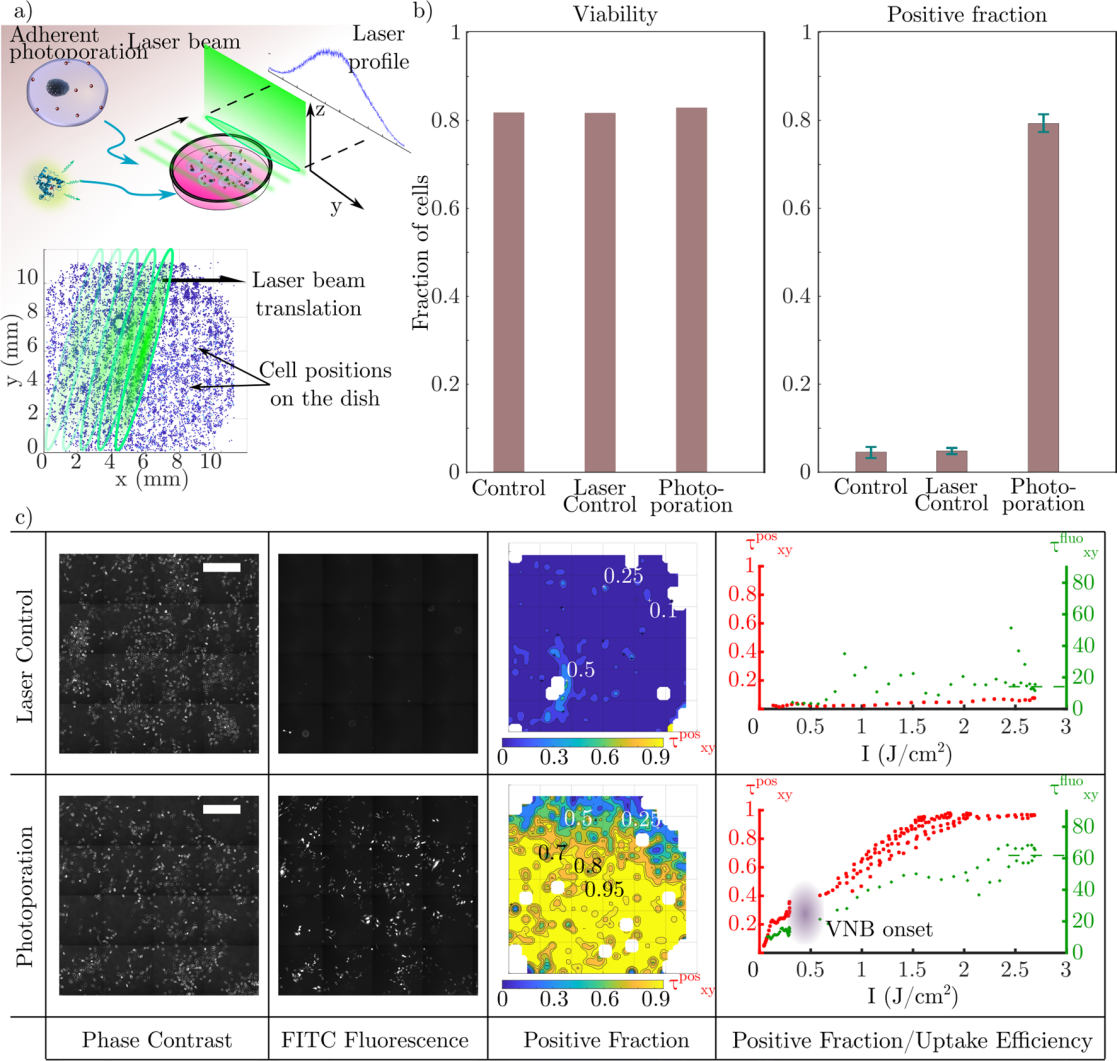
Adherent photoporation of HeLa cells induced by VNB collapse. **a** Scheme for adherent photoporation: Irradiation of a translating culture dish containing HeLa cells pre-incubated with AuNP by the elliptical nanosecond laser pulse. Permeabilization is monitored with the uptake of fluorescent FITC-Dextran ${10}~{\hbox {kDa}}$. The position of cells is compared to the laser intensity profile during the translation of the laser beam. **b** Global results of the adherent photoporation of HeLa cells with FITC-Dextran. Viability ($N^\text {after}/N^\text {before}$) and positive fraction ($N^\text {fluo}/N^\text {total}$) for the photoporation and control conditions are measured over the whole cell population of each sample. **c** Comparison between the laser control experiment (top) and the AuNP-mediated photoporation sample (bottom). Samples are imaged, reconstructed and divided into zones. In each zone, a local positive fraction is computed ($\tau _{xy}^\text {pos} = N_{xy}^\text {fluo}/N_{xy}^\text {total}$) and mapped over the reconstructed sample (example using a $I_\text {max} \simeq {2.6}~{\hbox {J cm}^{-2}}$). From left to right: Phase contrast images (scale bars ${500 }{\upmu {\hbox {m}}}$), FITC-fluorescence microscopy images, maps of the local positive fraction ($\tau _{xy}^\text {pos}$) computed from both images and finally the plot of the local positive fraction $\tau _{xy}^\text {pos}$ (red) and the mean uptake efficiency $\tau _{xy}^\text {fluo}$ (green) versus the intensity along the laser profile. $\tau _{xy}^\text {fluo}$ is measured with intra-cellular FITC fluorescence intensity. $\tau _{xy}^\text {pos}$ and $\tau _{xy}^\text {fluo}$ are eventually averaged along the direction perpendicular to the long axis of the laser beam to obtain the correlation with the different values of $I_\text {max}$ (resp. red and green) showing an onset between ${0.25}~{\hbox {J cm}^{-2}}$ to ${0.3}~{\hbox {J cm}^{-2}}$ for $\tau _{xy}^\text {pos}$, which can be related to the VNB onset

As an intermediate step, we first tried to photoporate adherent HeLa cells cultured in a glass bottom culture dish by scanning the dish through the elliptical beam as shown in Fig. 3a. The cells are photoporated with the optical set-up to validate the device for VNB generation (Fig. 2b,c). Prior to the irradiation of the culture dish, cells are pre-incubated with a suspension of gold nanoparticles at $8\cdot 10^7~\text {part.}{\hbox {mL}^{-1}}.$ [13]. The laser fluence used was $\simeq {2.6}~{\hbox {J cm}^{-2}}$ in order to generate VNBs around the ${70}~{\hbox {nm}}$ AuNPs used here [13]. The obtained photoporated sample is then imaged with fluorescence microscopy to assess membrane permeabilization through the uptake of FITC-Dextran ${10}~{\hbox {kDa}}$ (fluorescent probe). Fluorescence image analysis is performed to measure the average positive fraction over the whole population (Fig. 3b). The viability is measured by comparing the initial number of cells $N^\text {before}$ before laser treatment with the number of intact cells after irradiation and sample washing $N^\text {after}$. The irradiated samples with (photoporation) or without (laser control) AuNPs show $\simeq {80}{\%}$ viability. As a reference, the control sample with no AuNP and no irradiation shows $\simeq {85}{\%}$ viability. By comparing the intracellular fluorescence of the whole cell population, a global positive fraction is computed through the ratio of the number of fluorescent cells $N^\text {fluo}$ over the total number of cells retrieved after laser treatment $N^\text {total}$ ($\tau ^\text {pos} = N^\text {fluo}/N^\text {total}$). The AuNP photoporated sample shows a $\sim {80}{\%}$ positive fraction whereas the laser control (AuNP free sample) shows $\sim {5}{\%}$. This can be explained by the spontaneous uptake of FITC-Dextran during incubation by endocytic processes or by direct VNB generation due to the presence of nucleation points. These results are consistent with VNB photoporation [13].

During irradiation, not all cells receive the same laser pulse intensity due to the Gaussian laser profile along the long axis of the elliptical beam (Fig. 3a). To assess the effect of this difference on the delivery efficiency, cell positions are measured with respect to the laser beam orientation to infer the actual local fluence. Based on the same fluorescence images, the positive fraction is quantified for small areas of $\simeq {0.4}~{\hbox {mm}^{2}}$ (Fig. 3c - positive fraction). The local results are then correlated spatially with the fluence distribution of the laser beam. The correspondence is then obtained between the laser’s profile fluence applied locally and the local positive fraction $\tau _\text {xy}^\text {pos}$, retrieved from the mapped sample shown in the red plot in Fig. 3c - right column. Interestingly, this local spatially resolved study indicates a transition for low laser intensities. The local positive fraction increases rapidly before reaching a plateau at $\sim {20}{\%}$ for $I\simeq 0.1-0.25~{\hbox {J cm}^{-1}}$. This behavior can be explained by the increase of temperature with the intensity leading to better thermal permeabilization. The positive fraction takes off noticeably between 0.25 and ${0.3}~{\hbox {J cm}^{-2}}$ showing a possible onset of vapour nanobubble generation [34]. The AuNPs reach a high enough temperature to induce a phase change of the surrounding medium. Above these values, the contribution to the nanopores creation is consequently mainly mechano-acoustic.

Among other ways of discrimination between photo-thermal permeabilization and VNB-induced permeabilization, one can evaluate the uptake efficiency [13, 35]. We thus quantify the uptake efficiency $\tau _\text {xy}^\text {fluo}$, which is measured by the individual intra-cellular fluorescence of the positive cells (green plot in Fig. 3c - right column), increases with the fluence. At low intensities, where the main contribution is from the thermal regime, the fluorescence intensity varies slowly in the $\simeq 10-15~\text {a.u.}$ range. Considering that the thermal regime runs until ${0.3}~{\hbox {J cm}^{-2}}$, the obtained mean intra-cellular fluorescence is three times lower than the fluorescence obtained at VNB optimized conditions of $I_\text {max}\sim {2.6}~{\hbox {J cm}^{-2}}$ [13, 36]. Moreover, the increase of the fluorescence intensity with the fluence in the VNB regime shows a better uptake and hence an increase in nanopore effective surface (number or individual size) created with the AuNP irradiation, allowing more molecules to enter the cells.

The results presented for the photoporation of adherent HeLa cells are clearly consistent with what is reported in literature [36, 37]. They indicate that the chosen laser pulse energy is adequate for VNB generation in the elliptical laser beam.

### AuNP mediated Flow photoporation of HeLa cells in suspension

Next, we performed the flow photoporation of HeLa cells mixed by AuNPs as described in the Materials and Methods section below, without pre-incubation. During laser treatment, the flow rate is set such that each cell receives three laser pulses in total.

**Fig. 4:**
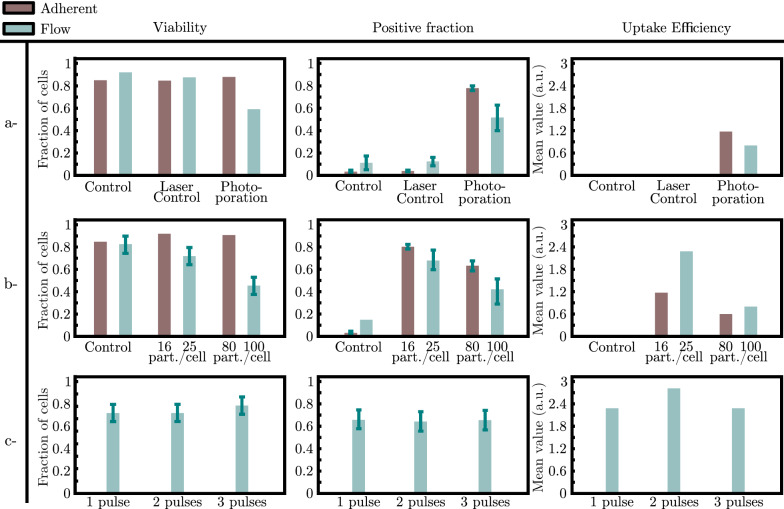
Comparison between adherent and flow photoporation of HeLa cells in presence of ${10}~{\hbox {kDa}}$ **FITC-Dextran.** Comparison of (i) the measured viability, (ii) the measured positive fraction and (iii) the measured uptake efficiency for the photoporation conditions with the intracellular fluorescence between controls and photoporation in both adherent and flow photoporation. **a** Comparison between adherent and flow photoporation and control conditions. **b** Comparison between two concentrations of AuNPs during photoporation in both adherent and flow photoporation with regard to the viability, the positive fraction and the uptake efficiency. **c** Comparison between 3 irradiations (1,2 and 3 pulses) of the cell-AuNP suspension during flow photoporation with regard to the viability, the positive fraction and the uptake efficiency

Flow photoporation results in ${56}{\%}$ positive cells compared to the adherent photoporation (${80}{\%}$). Both protocols were also run on non-adherent Jurkat cells yielding a positive fraction ${15}{\%}$ to ${20}{\%}$ lower than the ones obtained in the case of the HeLa cells. The latter results can be compared with flow (without laser and AuNPs) and laser (without AuNPs) control who yield ${10}{\%}$ and ${15}{\%}$ positive cells respectively whereas the laser control sample for the adherent cell photoporation shows a ${5}{\%}$ positive fraction (Fig. 4a). This difference can be attributed to the added contribution of shear stress along the microfluidic flow. Shear stress likely causes an increase of the number of permeabilized cells due to the stretching of the membrane. Moreover, the measured uptake efficiency shows a decrease in the concentration of FITC-Dextran inside the cytoplasm in the flow approach (Fig. 4a).

Viability of flow photoporated cells was markedly less at $54\%$ while it was $\ge {80}{\%}$ for the control conditions (Fig. 4a). A control consisting of a suspension of cells and nanoparticles flowing through the microfluidic chip without irradiation showed that the viability was ${80}{\%}$ fraction (data not shown). Additionally, in the main channel of the microfluidic chip, cells are subjected to fluid shear stress that we can estimate about ${15}{\hbox {\,dyn cm}^{2}}$. Moreover, the outlet channel has a radius 4 times smaller increasing the shear stress to reach $\sim {1000}{\hbox {\,dyn cm}^{2}}$. Such values of fluid shear stress in the outlet were reported to reduce the viability of malignant cells in microfluidic sized geometries [38]. Hence, we can assume that the shear stress is more influential than the presence of AuNPs and has an additive contribution with the photoporation to the reduced viability [39]. Moreover, this low viability can explain the lower positive fraction obtained in flow photoporation ($\sim {50}{\%}$) compared to the adherent cell configuration ($\ge {80}{\%}$) for the same fluence where $I_\text {max}\simeq {2.6}~{\hbox {J cm}^{-2}}$).

### Influence of the number of nanopores during adherent and flow photoporation

We continued to investigate the impact of the AuNP concentration on both the viability and the positive fraction after flow photoporation and adherent photoporation. In the case of the adherent photoporation, cells pre-incubated during ${30}~{\hbox {min}}$ with a $8\cdot 10^7~\text {part.}{\hbox {mL}^{-1}}$ AuNP suspension gives $\sim 8$ AuNPs attached per cell [13]. Considering the cell density in the cell suspension injected in the microfluidic chip (typically, $7-10\cdot 10^5\text {\,cells}{\hbox {mL}^{-1}}$), we assume that both cells and AuNPs are homogeneously distributed in the bulk liquid. In the case of the flow photoporation, AuNP concentrations of $2\cdot 10^7~\text {part.}{\hbox {mL}^{-1}}$ and $8\cdot 10^7~\text {part.}{\hbox {mL}^{-1}}$ were used. These values yield up to $\sim 25$ and $\sim 100$ AuNPs in a cell-equivalent volume respectively during the irradiation. In this experiment, cells were treated with a single laser pulse only.

For flow photoporation, the viability was $\sim {74}{\%}$ for the lowest AuNP concentration, and dropped to $\sim {40}{\%}$ for the highest concentration (Fig. 4b). Similarly, the positive fraction shows the same behavior through a decrease from $\sim {70}{\%}$ to $\sim {40}{\%}$ (Fig. 4b). Interestingly, reducing the concentration of AuNPs for the flow photoporation increases the positive fraction. It also improves viability thus reaching levels close to the adherent protocol (Fig. 4b). Presumably, increasing the number of nanoparticles increases the nanopores created in the cells’ membranes leads to cell death and a drop in the positive fraction.

We can then compare the uptake efficiency of both methods. The intra-cellular fluorescence measured for the different conditions of photoporation shows that for comparable number of nanoparticles ($\sim 10~\text {part.}/\text {cell}$ and $\sim 100~\text {part.}/\text {cell}$) the flow approach yields a better uptake efficiency (Fig. 4b).

For the same low concentration ($2\cdot 10^7~\text {part.}{\hbox {mL}^{-1}}$ giving $25~\text {part.}/\text {cell}$), we next investigate the effect of multiple laser pulses on the cells inside the microfluidic chip. This was achieved by decreasing the flow rate with respect to the fixed repetition rate of the pulsed laser. There were no appreciable differences in viability, positive fraction or uptake efficiency (Fig. 4c). This can be explained by the more adequate number of nanoparticles in the vicinity of the cell membrane after dilution. Unlike the ratio 100 : 1, with $\sim 25~\text {part.}/\text {cell}$, one pulse alters most of the AuNPs, rendering them ineffective for further laser irradiation [33, 40].

As a control to probe the effect of the number of nanopores created, we vary the number of AuNP attached to the cell membrane during the adherent photoporation (Fig. 4b). Cells are pre-incubated with two concentrations of AuNPs and rinsed prior to irradiation at the same optical conditions. Unlike the flow condition, the viability remains comparable to control values whether the cells are irradiated with $\sim 16$ or $\sim 80~\text {part.}/\text {cell.}$ Contrariwise, the positive fraction decreases with the concentration from $\sim {85}$ to $\sim {63}{\%}$. Interestingly, the $\sim 8$ and $\sim 16~\text {part.}/\text {cell.}$ yield very similar results for the positive fraction, the uptake efficiency and the viability.

The size of the nanopores created by the mechano-acoustic effects of AuNP-mediated photoporation depends on the size of the vapour nano-bubble generated. If we consider the effective surface of pores on the cell membrane, it depends on both the number of nanopores and their individual size. The former is related to the number of nanoparticles *N* as $\propto N^{1/2}$ and the latter to the nanobubble’s radius which varies following $\propto I^{1/3}$ with *I* the laser pulse intensity [11, 41–43]. The effective pore size for the cell to repair is thus more influenced by the number of the VNB generation sites than by the intensity, the latter occurring along the laser beam’s major axis [44, 45].

The remark above may indicate longer repair times for the adherent cells photoporated with a higher AuNP concentration in our adherent photoporation experiment presented earlier. Because diffusion times depend on the nanopore radius, the last longing nanopores can cause the intracellular FITC-Dextran to diffuse out of the cytoplasm eventually, explaining the drop in the positive fraction measured [44]. Consistently, the intracellular fluorescence is lower in the case of the adherent cell photoporation with a high AuNP concentration than the low concentration experiment.

If we consider the possible longer repair time transposed to the flow configuration, the shear stress induced by the flow within the microfluidic chip can cause supplementary damage to the cell membranes subsequently to the laser treatment [26, 46]. This explains the drop in in the viability, unlike the adherent approach, along the positive fraction [42, 47]. This same drop in viability can be a consequence to the low positive fraction obtained.

It was interesting to see that the amount of FITC-Dextran delivered in cells via flow photoporation was higher than for photoporation on adherent cells. This may be due a difference in the permeabilization process.

The permeabilization process is initiated through the optical excitation of plasmonic nanoparticles to achieve VNB generation. The bubble dynamic at the nano/micro scale can induce membrane permeabilization in three ways. First, through the oscillation of the bubble radius and the resulting shear stress caused by the fluid motion [48–50]. Secondly, upon the collapse of the bubble, a shock wave can be emitted and induce a mechanical stress creating the nanopores. Thirdly, the pores can result from liquid nanojets [51]. If the bubble dynamics happen close to a solid boundary, for example a cell membrane, the collapse eventually causes the generation of a liquid nanojet whose orientation depends on the mechanical properties, namely Young’s modulus. This nanojet emission can occur if the distance separating the nanobubble is comparable to its maximum radius at expansion and induce the membrane permeabilization [47, 52, 53].

In the case of attached nanoparticles to the cell membrane, it is reported that the collapse does not induce a nanojet since the bubble is of hemispherical shape [54, 55]. Therefore, for adherent photoporation, liquid nanojets are not expected to contribute substantially to the delivery process.

The protocol for flow photoporation, on the other hand, does not favor the nanoparticles attachment. This was confirmed using confocal laser scanning microscope on two samples containing a working suspension of HeLa cells and AuNP where the first one was simply incubated whereas the second was harvested after passage through the microfluidic device ($37~\text {part}/\text {cell}$ for the incubated cells *vs* $20~\text {part}/\text {cell}$ the harvested cells). Given the laminar and poorly diffusive flow conditions (Reynolds number of 4 and Peclet number of 20), the AuNP attachment is similar for the three flow rate values presented. Consequently, most AuNPs will be dispersed around the cells at some distance from the cell membrane. With this assumption, the nanobubble and the cell membrane are separated with a liquid layer favoring nano-jet formation [56]. One of the advantages of the nano-jet induced permeabilization is the loading speed obtained by advection compared to diffusion [32]. Additionally, nanojets generate nanopores of smaller sizes. We can suppose that repair times are shorter, limiting the release of FITC-Dextran outside the cytoplasm. This improved delivery may explain why the average uptake efficiency for the flow photoporation was twice as important as for the case of adherent photoporation (Fig. 4b).

The comparative uptake efficiency can be explained with the nanojet generation as reported in the literature, thus optimizing the uptake process as the convection induced is. This can be considered an indirect evidence of the poor adsorption of the AuNP to the cells’ membranes during the flow photoporation approach of the suspension in comparison with the AuNP-attached adherent photoporation. This limited adsorption opens the possibility of separating cells and AuNPs which remains after irradiation during the sample preparation.

## Conclusions

We present an optofluidic device composed of a microfluidic chip achieving a two stage flow focusing. Adherent HeLa cells are suspended and mixed with a suspension of gold nanoparticles to limit adsorption prior to irradiation with a nanosecond laser pulse inside the microfluidic chip. The optical excitation is characterized through the photoporation of adherent HeLa cells pre-incubated with AuNP to confirm the generation of vapour nanobubble around the AuNP. The results obtained are used to assess the flow photoporation protocol by comparing the positive fraction, the viability and the uptake efficiency. For different photoporation conditions (number of pulses applied, number of particles per cells), the output of the FITC-dextran uptake shows the generation the VNB in the microfluidic flow conditions is achieved. The flow approach can yield up to $\sim {70}{\%}$ positive fraction and $\sim {80}{\%}$ viability. With results comparable to the adherent photoporation approach, the flow photoporation eliminates some barriers like being applicable to non adherent cell lines. In addition,it appears to take advantage of a flow jet generation during the collapse of the vapour nanobubble, improving the uptake efficiency of the cargo. Moreover, circumventing the pre-attachment step of the nanoparticles to the cells prior to irradiation allows a subsequent filtration of AuNP residues and debris due to the laser irradiation. Other couplings can be achieved with labeling steps, sorting or fluorescence triggered systems. The versatility of the present apparatus can be improved while benefiting from the benefits inherent to microfluidics such as controlled conditions of irradiation and sample manipulation. Finally, throughput is another key-parameter in any transfection process. The current apparatus allows a throughput of $10^3-5\cdot 10^4~\text {cell.}{\hbox {min}^{-1}}$ and is limited mainly by the current laser’s repetition rate. With microfluidics, the flow rates can be adapted instantaneously to the laser’s frequency and the cell events’ frequency, especially in the case of targeting isolated cells [57]. These findings make of this method a bridge toward labeled single cell or high throughput transfection, circumventing thus barriers to the translation and clinical deployment of emerging cellular therapies.

## Materials and methods

### Microfluidic chip fabrication and process

Due to optical constraints (laser induced damage, light transmission) and high pressure, microfluidic chips were fabricated on a Glass-Silicon-Glass basis with perpendicular PTFE inlets. ${1}~{\hbox {mm}}$ thick 3 inch glass wafer and ${100}~{\upmu \hbox {{m}}}$ thick silicon wafer of same diameter are assembled using anodic bonding. The Silicon face is patterned following optical lithography techniques where a layer of spin-coated resist is insulated with UV light through a printed plastic mask. The patterned surface is then deep reactive-ion etched according to a custom Bosch process. After cleaning and pinching flow conduits with a ${355}~{\hbox {nm}}$ laser with $\hbox {ns}$ pulse, another anodic bonding is performed to seal the chip with an identical glass wafer.

The microfluidic design resembles the one for hydrodynamic focusing with a central inlet, two set of symmetrical lateral inlets and 3 outlets to perform further separation. Flow control is performed using pressure inputs servo-controlled by flow sensors.

Tubings upstream were adapted to the microfluidic apparatus and to boost cell dissociation in the event of clump formation in the reservoir during the experiment.

### Optical design

${70}~{\hbox {nm}}$ spherical AuNPs with a SPR peak at ${542}~{\hbox {nm}}$ are irradiated with Nd:YAG Q-switch laser generating ${5}~{\hbox {ns}}$ wide pulses at a ${10}~{\hbox {Hz}}$ repetition rate emitting at ${532}~{\hbox {nm}}$. The beam is filtered to remove residual wavelengths and polarizations and shaped through a beam expander and a cylindrical lens to obtain an elliptical laser beam on the sample to fit the microfluidic chip’s geometry.

The laser beam has typical dimensions of $\simeq {2}~{\hbox {cm}}\times {0.1}~{\hbox {mm}}$ and power is measured before expansion to control the ${2.5}~{\hbox {J cm}^{-2}}$ fluence using an optical powermeter. The optical components were chosen to withstand high-power laser pulses.

The chip is illuminated with a white LED and visualized using a CMOS camera (Hamamatsu) at $\sim 35$ fps. The number of cells per unit time is checked both on camera (high speed streaming) and using a He-Ne laser monitored by a photodiode to measure the number of cells passing through the focused beam in real time. This optical barrier is controlled with a LabView virtual instrument.

### Sample preparation for adherent photoporation

During this study, HeLa WT was the main cell line tested and was cultured in high glucose Dulbecco’s Modified Eagle Medium (DMEM) supplied with ${10}{\%}$ Fetal Bovine Serum (FBS), ${1}{\%}$ L-Glutamine and ${0.4}{\%}$ Penicillin-Streptomycin. Prior to photoporation, cells are sub-cultured in P35 glass bottom dishes and pre-incubated with a suspension of AuNP in complete medium for ${30}~{\hbox {min}}$ at ${37}^{\circ }{\hbox {C}}$. After a rinsing process with Dulbecco’s Phosphate Buffered Saline without calcium or magnesium (DPBS), complete pre-heated medium containing ${10}~{\hbox {kDa}}$ FITC-dextran at ${2}{\hbox {mg mL}^{-1}}$ is added. The sample is then placed on the sample holder attached to a mobile stage. Depending on the purpose,the Gaussian (for local transfection) or the elliptical beam (for treating the entire sample) is used to swipe the dish.

For efficiency purposes, laser, stage and acquisition parts were interfaced with a computer using LabView and NI’s PCIe 6323 Xseries. To mimic the behavior the laser would have during flow experiments, the cadence of operation was set to the one of the laser’s ${10}~{\hbox {Hz}}$ repetition rate, to perform stage translation, laser illumination and image acquisition.

Given that the VNB-induced membrane pores are transient and repaired in few tens of seconds, the sample is rinsed several minutes after laser treatment twice with DPBS to evacuate the excess of FITC-dextran and fresh medium is added for further imaging.

### Sample preparation for flow photoporation

The flow photoporation of HeLa WT cells mixed with AuNPs without pre-incubation, through a microfluidic chip was performed as follows. HeLa WT cultivated in T75 flasks are trypsinized and centrifuged in complete medium (DMEM). The pellet is then resuspended in Dulbecco’s Phosphate Buffered Saline without calcium or magnesium (DPBS) with thorough trituration to avoid clump formation. Prior to connection to the microfluidic chip, a volume of AuNP suspension at a concentration of $8\cdot 10^7~\text {part.}{\hbox {mL}^{-1}}$ is added to the cell suspension. The tube is then plugged to the microfluidic inlet.

To guarantee minimum medium, the remaining inlets contain DPBS supplied with nominal glucose concentration as buffer. To assess pore creation, ${10}~{\hbox {kDa}}$ FITC dextran is dissolved in the buffers except the one carrying cells to minimize passive embedding of the macromolecule. During photoporation, The mixture is spatially confined in the main channel with the flow focusing geometry and irradiated with the elliptical laser beam at a ${10}~{\hbox {Hz}}$ repetition rate as was shown earlier in Fig. 2a).

Once the monitored number of cells or the volume reaches the target value, the treated sample is retrieved from the outlet reservoir and fresh pre-heated medium is added to dilute the surrounding fluorescence and provide the sample with nutrients. The samples are then centrifuged twice to eliminate the supernatant and the pellet is re-suspended in complete medium and transferred to a glass bottom culture dish for further imaging.

Prior to the re-plating, images of the samples’ pellets are taken in suspension chambers ($\simeq {2.5}~{\upmu {\hbox {L}}}$ from ${100}~{\upmu {\hbox {L}}}$ pellet) to assess the number of cells retrieved (viability) and their fluorescence.

### Image analysis and Transfection assessment

Photoporation assessment - for adherent and flow - is made by imaging FITC-Dextran intra-cellular fluorescence on an inverted optical microscope Ti Eclipse 2000 Nikon using a S Plan Fluor 20x dry objective with a 0.45 numerical aperture. Samples are imaged ${24}~{\hbox {h}}$ after irradiation in both protocols to insure re-attachement of suspended cells in the flow approach. Cells are treated with a Hoechst stain prior to imaging to stain the nuclei for easier cell detection during image segmentation steps.

Each sample is imaged for phase contrast, DAPI fluorescence for the nuclei staining and GFP-FITC for dextran intake to quantify the intra-cellular fluorescence and evaluate in-take efficiency. Using Nikon’s Perfect Focus System, focused images are taken for different positions.

Image analysis is run with a homemade script on Matlab to achieve object detection, segmentation and feature extraction (size, circularity and fluorescent signal intensity). The same script extract viability, positive fraction and population growth to completely quantify the photoporation performances.

## Data Availability

The data that support the findings of this study are available from the corresponding author upon reasonable request.

## Abbreviations

AuNP: Gold NanoParticle
DMEM: Dulbecco’s modified eagle medium
DPBS: Dulbecco’s phosphate buffered Saline
FBS: Fetal bovine serum
FITC: Fluorescein isothiocyanate
SPR: Surface plasmon resonance
VNB: Vapour nanobubble
WT: Wild type
